# Geographic Availability of Assistance Dogs: Dogs Placed in 2013–2014 by ADI- or IGDF-Accredited or Candidate Facilities in the United States and Canada, and Non-accredited U.S. Facilities

**DOI:** 10.3389/fvets.2019.00349

**Published:** 2019-10-09

**Authors:** Sandra Walther, Mariko Yamamoto, Abigail P. Thigpen, Neil H. Willits, Lynette A. Hart

**Affiliations:** ^1^School of Veterinary Medicine, University of California, Davis, Davis, CA, United States; ^2^Teikyo University of Science, Uenohara, Japan; ^3^Department of Population Health and Reproduction, School of Veterinary Medicine, University of California, Davis, Davis, CA, United States; ^4^Department of Statistics, University of California, Davis, Davis, CA, United States

**Keywords:** assistance dogs, service dogs, autism service dogs, mobility service dogs, hearing dogs, psychiatric service dogs, seizure assistance dogs, diabetes alert dogs

## Abstract

Assistance dogs' roles have diversified to support people with various disabilities, especially in the U.S. Data presented here are from the U.S. and Canada non-profit facilities (including both accredited and candidate members that fulfilled partial requirements: all here termed “accredited”) of Assistance Dogs International (ADI) and the International Guide Dog Federation (IGDF), and from non-accredited U.S. assistance dog training facilities, on the numbers and types of dogs they placed in 2013 and 2014 with persons who have disabilities. ADI categories of assistance dogs are for guide, hearing, and service (including for assistance with mobility, autism, psychiatric, diabetes, seizure disabilities). Accredited facilities in 28 states and 3 provinces responded; accredited non-responding facilities were in 22 states and 1 province (some in states/provinces with responding accredited facilities). Non-accredited facilities in 16 states responded. U.S./Canada responding accredited facilities (55 of 96: 57%) placed 2,374 dogs; non-accredited U.S. facilities (22 of 133: 16.5%) placed 797 dogs. Accredited facilities placed similar numbers of dogs for guiding (*n* = 918) or mobility (*n* = 943), but many more facilities placed mobility service dogs than guide dogs. Autism service dogs were third most for accredited (*n* = 205 placements) and U.S. non-accredited (*n* = 72) facilities. Psychiatric service dogs were fourth most common in accredited placements (*n* = 119) and accounted for most placements (*n* = 526) in non-accredited facilities. Other accredited placements were for: hearing (*n* = 109); diabetic alert (*n* = 69), and seizure response (*n* = 11). Responding non-accredited facilities placed 17 hearing dogs, 30 diabetic alert dogs, and 18 seizure response dogs. Non-accredited facilities placed many dogs for psychiatric assistance, often for veterans, but ADI accreditation is required for veterans to have financial reimbursement. Twenty states and several provinces had no responding facilities; 17 of these states had no accredited facilities. In regions lacking facilities, some people with disabilities may find it inconvenient living far from any supportive facility, even if travel costs are provided. Despite accelerated U.S./Canada placements, access to well-trained assistance dogs continues to be limited and inconvenient for many people with disabilities, and the numerous sources of expensive, poorly trained dogs add confusion for potential handlers.

## Introduction

With little monitoring to track changes in assistance dog placements over time, assistance dogs' roles have rapidly diversified to support people with various disabilities, especially in the U.S. since passage of the Americans with Disabilities Act (1). This U.S. legislation and its enabling regulations assure reasonable accommodation, which includes public access for a person with an assistance dog, sometimes termed service dog (2). Emphasizing that the dog performs tasks that assist with the person's disability, the U.S. uses the inclusive term “service dog,” whereas internationally, “assistance dog” is the inclusive term that includes all dogs fulfilling assisting roles for persons with disabilities (and is the term primarily used here). Lacking any centralized registration process, not requiring any specific accreditation verifying the training of the dogs, and allowing people to train their own assistance dogs, the U.S. has no system for monitoring the numbers or types of assistance dogs that are working and makes it easy for new facilities or someone with a disability to train such dogs. Thus, numerous informal training procedures or facilities exist in the U.S. In contrast, some other countries specify and limit who is qualified to train assistance dogs for public access. For example, Japan (2) and Taiwan (3) have a centralized method for tracking assistance dogs.

Legislation and regulations in the U.S. assure persons with disabilities the right to have public access with their assistance dogs that perform tasks related to the person's disability (4). Although it is required that the dog be trained in these tasks, the method and source of the training are unspecified and no certification process or special identification is required for the assistance dog or its handler. With this permissive framework, both the numbers and types of assistance dogs have sharply increased in recent decades, particularly in the U.S.; placements in Europe show a similar trend that is less rapid (5). Also, the types and body sizes of dogs used in assistance work are changing and now include a wide range of purebred and mixed breed dogs acquired from various sources, with many small as well as larger dogs serving in the various assisting roles (5, 6). It adds confusion that in the U.S., emotional support animals for people with disabilities are recognized by Housing and Urban Development for access with the handler to housing (7–9) and by the U.S. Department of Transportation for access with the handler to air travel (10); these animals are not required to perform tasks and are not being addressed in this paper.

Assistance Dogs International (ADI) categorizes the roles of assistance dogs as guide, hearing, and service; the roles of service dogs include assistance for mobility, autism, seizures, psychiatric symptoms, and medical alert (11). Like the International Guide Dog Federation [IGDF; (12)], ADI accredited facilities are required to be non-profit, and must fulfill the extensive requirements of ADI Standards (5). As some examples, facilities must assure the long-term support of clients and dogs, and dogs are expected to be people oriented, and not aggressively protective. Accredited facilities also are required to have a strong track record of successful placement of human-assistance dog teams. Facilities that are seeking to become accredited and that already fulfill some of the requirements can become candidate facilities.

Additionally, ADI provides facilities with specific standards for training and placement of assistance dogs for veterans with military-related PTSD (13), requiring that the dog facilitate friendly public interaction with the veteran and have training based on praise and positive affect, and that the veteran-service dog team be supported by at least two individuals, such as family members. Candidate and accredited facilities placing these dogs with veterans are required to have a licensed mental health professional available, and address issues of suicide and anger management.

Historically in the U.S., guide dog facilities were established from 1929 through the 1950s; subsequently numerous mobility service dog and a few hearing dog facilities were founded from 1973 through the 1990s (5). More recently, additional new facilities were established, contributing to the growth of dogs' roles for assistance with psychiatric, autistic, and medical alert needs. A similar pattern occurred in Europe, with the expansion of numerous mobility service dog facilities and one large hearing dog facility beginning in the 1980s. Facilities were established outside U.S./Canada and Europe beginning in 1957; the large majority of these facilities still place solely guide dogs (5).

With the proliferation of assistance dogs in the U.S., along with increasing numbers of emotional support animals that are allowed access in housing and air transport (7–10), and growing use of therapy dogs in animal-assisted interventions, social conflict has arisen and confusion has increased regarding the varied roles of dogs and their legally allowed public access. Societal conflict primarily has focused on animals in airplanes, leading airlines to create new policies regarding animals in the airplane cabins (14, 15). Legislators have sought solutions (16), and the American Veterinary Medical Association (AVMA) has endeavored to provide accurate information (17), develop clarifications, and broker solutions for revised policies or new legislation. Concern has grown that some assistance dogs or emotional support animals have inappropriate behavior, and that purported assistance dogs may be fraudulently labeled by their handlers if the dogs lack appropriate behavior or do not perform tasks related to the handler's disability.

Some states have legislation strengthening protection of public access with assistance dogs and assuring access to people with assistance dogs in training, including punishment for interfering with or injuring dogs, e.g., California and Florida [summarized in 2006 worldwide by ADI, (18)]. Some pushback limiting assistance dogs has come from legislation in other states. Also, the U.S. Army and Veterans Administration, appreciating the specified training requirements of ADI, require that their clients acquire assistance dogs from facilities accredited by ADI and will not reimburse expenses for dogs acquired from other sources (19, 20). Yet, persons seeking to acquire an assistance dog may not be familiar with ADI and the training and placement process involved. They may lack knowledge of how to assess a non-accredited facility placing dogs and may be vulnerable to opportunists. Finding access to this essential information on well-trained dogs can be challenging in the U.S.; this was the case when studied among people with visual and other physical disabilities in Japan (21, 22). Many facilities that place dogs have long waiting lists, adding frustration to the process of expeditiously acquiring a dog. While handlers in the U.S. are allowed to train their own assistance dogs, supportive resources that are economical and effective for this approach may not be easy to find. Some private dog trainers sell trained assistance dogs for very high prices, but then when the dogs do not always perform in the role that was promised (23), the person with a disability who needs a canine partner has no recourse.

The objective of this survey was to assess current geographic patterns of placements of assistance dogs, focusing on the states of the U.S. and the provinces of Canada, where the numbers and roles of these dogs have been expanding rapidly.

## Materials and Methods

For this study, all U.S. and Canada facilities associated with ADI or IGDF were contacted up to three times by e-mail and sometimes telephone, if requested by the facility, concerning the numbers and roles of dogs they placed in 2013 and 2014 with persons who have disabilities, requesting that the facilities complete a brief survey. Both accredited facilities and candidate facilities that are seeking to become accredited and that have already fulfilled some of the accreditation requirements were contacted; these all generally are termed accredited in subsequent text. Among these facilities, 55 out of 96 (57%) facilities responded and provided information on their placements of 2,374 dogs.

We also e-mailed a survey to all non-accredited U.S. facilities listed online. The initial list of 170 facilities was developed by searching: assistance dogs, service dogs, mobility service dogs, seizure response dogs, diabetes alert dogs, autism service dogs, PTSD dogs, and psychiatric service dogs; lists of facilities posted online also were gathered. Facilities also appearing on ADI or IGDF lists were deleted, as well as duplicates. Email invitations were successfully transmitted to 133/170 facilities; 37/170 (22%) bounced back from failed addresses, reflecting turnover. Among the 133 invited non-accredited U.S. facilities, the response rate by 22 facilities was 16.5%, reflecting their placements of 797 dogs. Two reminder emails were sent to all non-respondents.

We assessed placements of the dogs for the various roles throughout the U.S. and Canada as related to the facility's year of establishment. The survey was distributed to facilities worldwide and some results were previously published (5), whereas this study focuses on the specific results from North America. The survey included the following questions: year that the facility started producing dogs; the numbers and roles of dogs placed in 2013 and 2014; the total number of assistance dogs that were placed each year; numbers of guide, hearing, mobility service, seizure response, autism service, diabetic alert, and psychiatric service dogs placed; the breeds of dogs used; the sources of the dogs (breeding within the program, outside breeders, clients' pets, shelters, or other sources); and the duration of team training in which a new handler is taught to work with the canine partner.

Data were analyzed using chi-squared tests of independence between particular categorical variables, including the relationships between types of assistance dogs, geographical regions, accreditation, and the sources of the dogs.

## Results

### Geographic Distributions of Facilities Placing Dogs in Various Roles

Accredited facilities in 28 states and 3 provinces responded; accredited non-responding facilities were in 22 states and 1 province (some in states/provinces with responding accredited facilities). Non-accredited facilities in 16 states responded.

**Figure 1:**
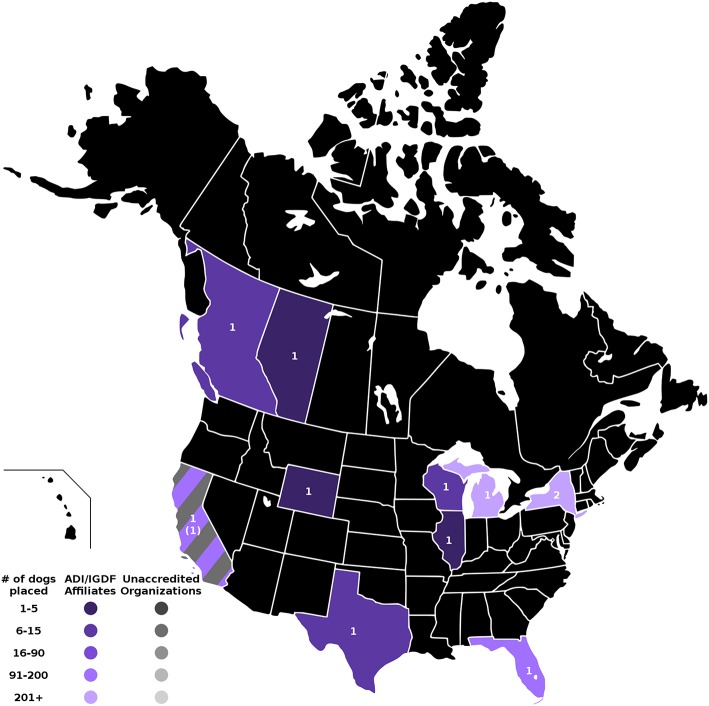
Guide dog facilities and placements in the U.S. and Canada. Digital numbers in states and provinces indicate numbers of responding facilities, with non-accredited facilities in parentheses. Color coding for each state or province indicates the range of numbers of guide dogs placed in 2013 and 2014, with accredited facilities represented in color, and non-accredited facilities in grayscale.

**Figure 2:**
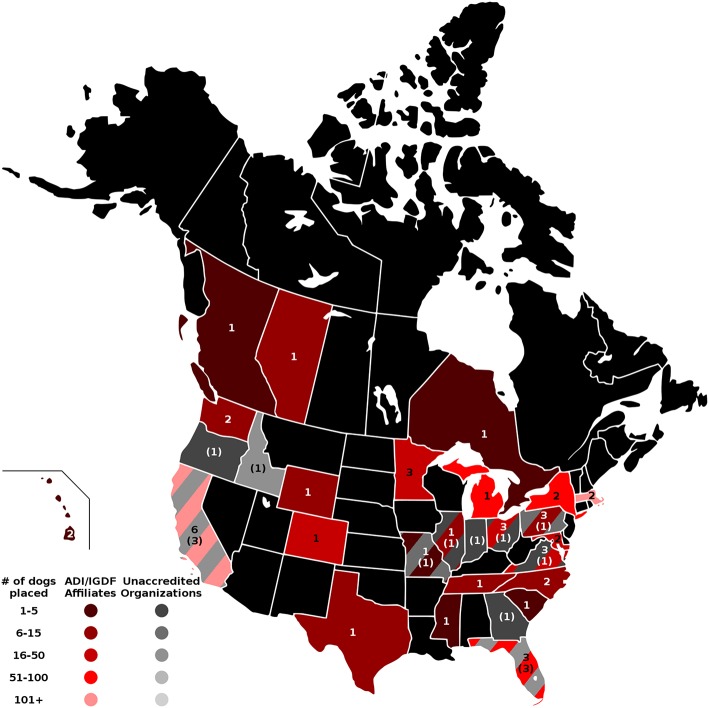
Mobility service dog facilities and placements in the U.S. and Canada. Digital numbers in states and provinces indicate numbers of responding facilities, with non-accredited facilities in parentheses. Color coding indicates the ranges of total numbers of mobility service dogs placed in 2013 and 2014 for accredited and non-accredited facilities.

**Figure 3:**
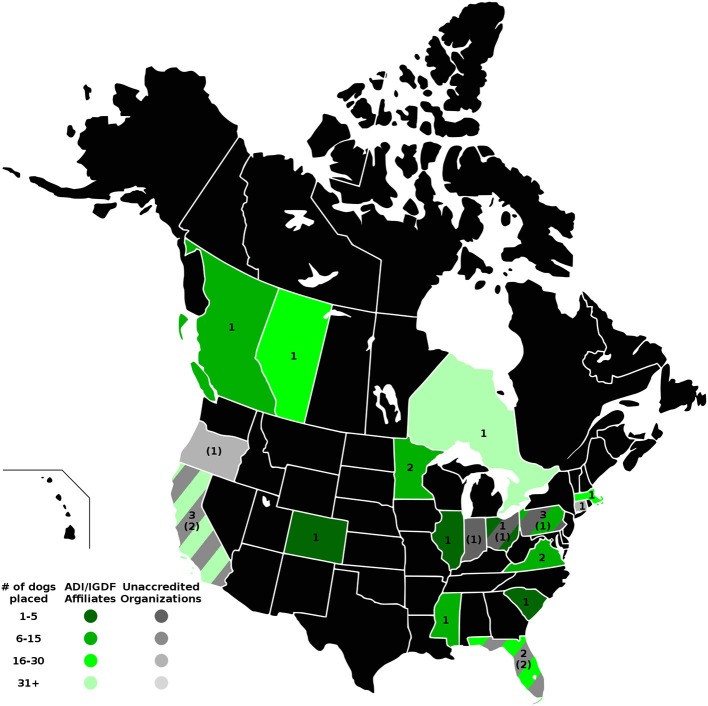
Autism service dog facilities and placements in the U.S. and Canada. Digital numbers in states and provinces indicate numbers of responding facilities, with non-accredited facilities in parentheses. Color coding indicates the ranges of total numbers of autism service dogs placed in 2013 and 2014 for accredited and non-accredited facilities.

**Figure 4:**
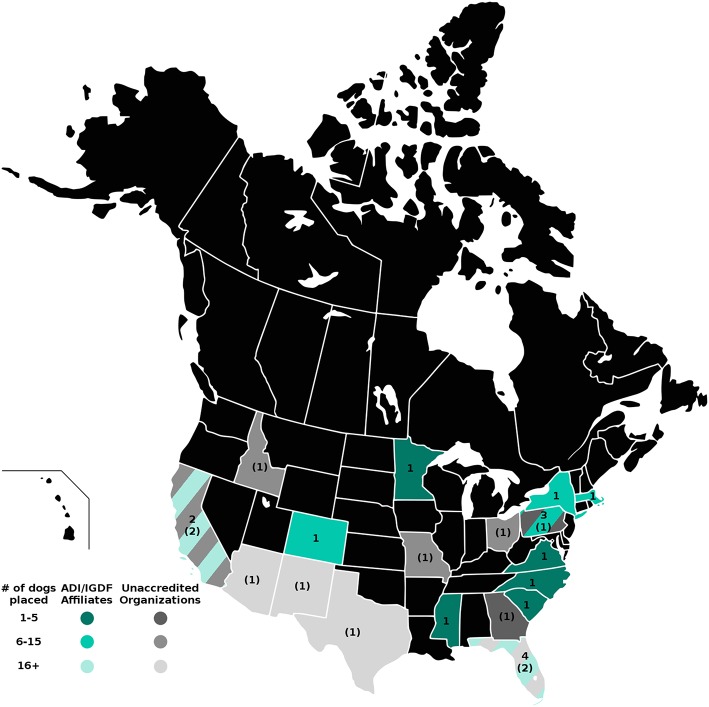
Psychiatric service dog facilities and placements in the U.S. and Canada. Digital numbers in states and provinces indicate numbers of responding facilities, with non-accredited facilities in parentheses. Color coding indicates the ranges of number of psychiatric dogs placed in 2013 and 2014 by accredited and non-accredited facilities.

**Figure 5:**
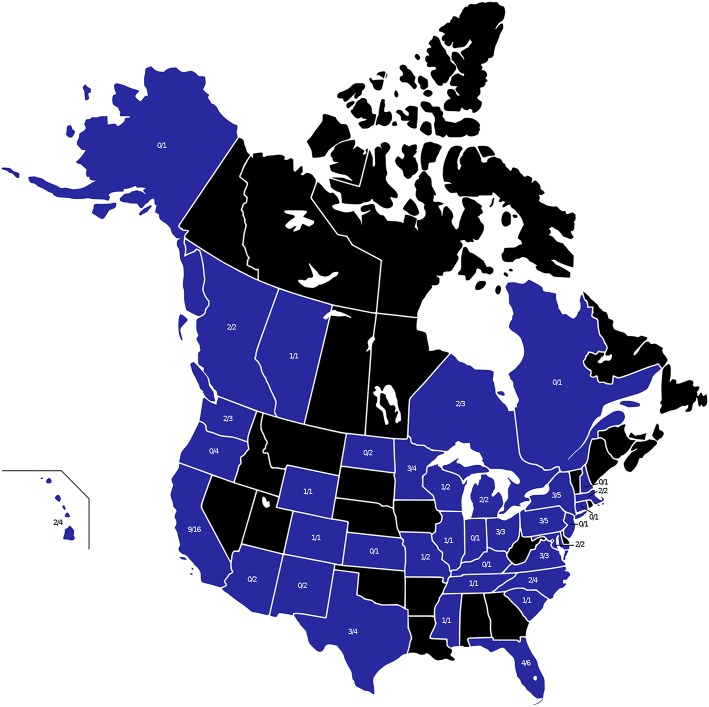
All states and provinces with ADI and/or IGDF facilities at the time of the survey are shown in blue. The numerator indicates the number of responding facilities and the denominator shows the total number of ADI and/or IGDF facilities at the time of the survey.

**Figure 6:**
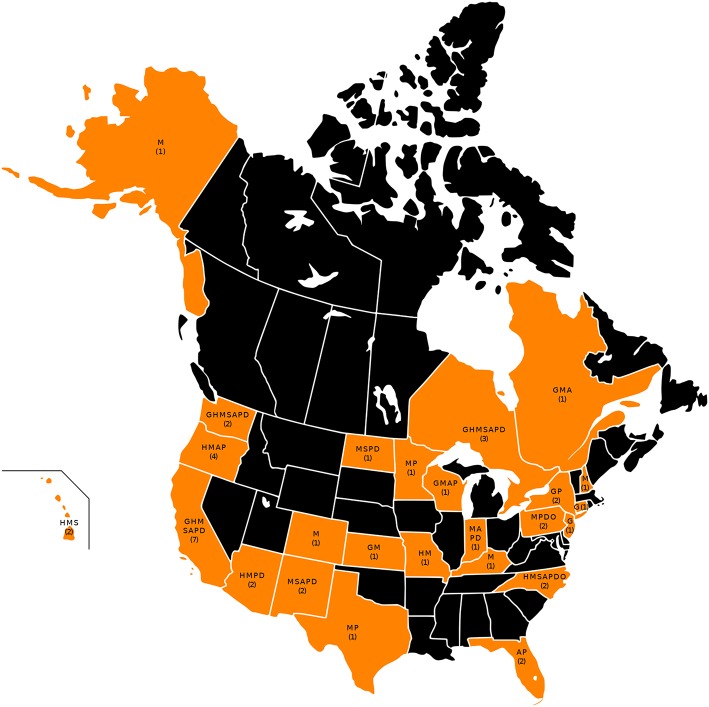
All states and provinces with non-responding ADI and/or IGDF facilities at the time of the survey are shown in orange. The number of these non-responding facilities is shown on orange states and provinces. The letters indicate the types of dogs placed by these facilities in each state, according to their websites, listed in the order used in the survey. Key: G, guide; H, hearing; M, mobility; S, seizure; A, autism; P, psychiatric; D, diabetes.

The four maps in Figures 1–**4** represent the distributions of responding service dog organizations from the U.S. and Canada placing: guide dogs; mobility service dogs; autism service dogs; and psychiatric service dogs. The plain numbers and colors represent the numbers of responding ADI/IGDF accredited facilities in U.S. or Canada, while the numbers in parentheses and the grayscale represent the numbers of responding unaccredited U.S. facilities. For each role of dog, the approximate numbers of dogs placed by the responding facilities in each state during the 2 years is indicated by the shaded colors shown on the figure legend. For these and subsequent figures, Figures 1–**6**, the same numerical information also is provided in Supplementary Tables. This will accommodate anyone to more easily see the actual numbers and interpret the data that are provided here.

#### Guide Dogs

Placements of guide dogs in the U.S./Canada were very numerous (accredited facilities, *n* = 918; non-accredited facilities, *n* = 3), with the number of placements of guide dogs by 11 accredited facilities similar in numbers to placements of mobility dogs in the same period. Facilities training and placing guide dogs consistently placed primarily guide dogs, often in somewhat large numbers. Some facilities placing guide dogs occasionally produced dogs trained to fill other roles, but in much fewer numbers. Guide dogs were the first type of service dog placed in the U.S., beginning in 1929.

As shown in Figure 1, accredited facilities placing guide dogs only responded in 8 states and 2 provinces. Facilities in the states of Michigan and New York placed the most, over 200 guide dogs per facility, followed by California and Florida, then Wisconsin, Texas, and British Columbia. Responding facilities in Illinois, Wyoming, and Alberta each placed 5 or fewer guide dogs. New York was the only state with a response from more than one accredited facility placing guide dogs. There was an additional response from one non-accredited facility in California that had placed a few guide dogs.

#### Mobility Service Dogs

The total number of mobility service dogs placed was 1054 (accredited facilities, *n* = 943; non-accredited *n* = 111). This was similar to the number of guide dog placements from U.S./Canada accredited facilities, but these dogs were placed by far more facilities (*n* = 60), both accredited (*n* = 45) and non-accredited (*n* = 15). Service dogs for mobility were not always the most numerous type of dog placed by these facilities. Historically, mobility service dogs were the second earliest type of service dog placed by these facilities, the first facility producing them appeared in 1973. As shown in Figure 2, responses were received from accredited facilities placing mobility dogs in 21 states and 3 provinces. Responses were received from non-accredited facilities in 11 states. California and Florida placed a large number of dogs. Numerous states had responses from both accredited and non-accredited facilities placing mobility service dogs, including California, Florida, Illinois, Missouri, Ohio, Pennsylvania, and Virginia. Georgia, Idaho, Indiana, and Oregon each had only one responding non-accredited facility.

No guide dog or mobility service dog facilities responded from Alabama, Alaska, Arizona, Arkansas, Connecticut, Iowa, Kansas, Kentucky, Louisiana, Maine, Montana, Nebraska, Nevada, New Hampshire, New Mexico, North Dakota, Oklahoma, South Dakota, Utah, Vermont, and West Virginia (42% of states). In Canada, no guide dog or mobility service dog facilities responded from Labrador, Manitoba, the Maritime Provinces, Northwest Territory, Newfoundland, Nunavut, Quebec, Saskatchewan, and Yukon, but one accredited facility for mobility dogs responded in each of the following provinces: British Columbia, Alberta, and Ontario.

#### Autism Service Dogs

Placements of autism service dogs were the third most numerous type of dog placed by accredited facilities for the 2 years in U.S./Canada (*n* = 205 dogs) and also third for U.S. non-accredited facilities (*n* = 72 dogs). The number of autism service dogs placed increased by 16% from 2013 to 2014 in U.S./Canada for accredited facilities. Four U.S. accredited facilities listed autism service as their primary placements. In the U.S., five accredited mobility service facilities established in the 1970–1980s listed autism service dogs as their second or third most numerous type placed. Among responding non-accredited facilities, the oldest facility, established in 1984, placed all seven types of dogs, with autism service their fifth most numerous type. Five other non-accredited facilities placed primarily autism service dogs. As shown in Figure 3, there were multiple responses from both accredited and non-accredited facilities in California, Florida, Pennsylvania, and Ohio. Minnesota and Virginia each had 2 accredited facilities, and remaining states and provinces had a single accredited or non-accredited facility. Altogether, 14 states (11 with accredited facilities; 7 with non-accredited facilities) had responses from facilities producing autism service dogs (accredited facilities *n* = 18; non-accredited facilities *n* = 9). As with mobility service dogs, there was one response from an accredited facility placing autism service dogs in each of the following Canadian provinces: British Columbia, Alberta, and Ontario.

#### Psychiatric Service Dogs

Placements of psychiatric service dogs by accredited facilities were fourth most common in U.S./Canada (*n* = 119 dogs), surpassing hearing dog placements. Among reporting non-accredited U.S. facilities, psychiatric dogs accounted for the most placements (*n* = 526 dogs). As shown in Figure 4, while only 11 states had responses from accredited facilities placing psychiatric service dogs, 10 states had responses from non-accredited facilities placing psychiatric service dogs. Hence, over one-third of responding states had responses from facilities placing psychiatric dogs. Once again, California and Florida each had responses from multiple accredited and non-accredited facilities. Colorado, Massachusetts, Minnesota, Mississippi, New York, North Carolina, South Carolina, and Virginia each had one response from an accredited facility. Arizona, Georgia, Idaho, Missouri, New Mexico, Ohio, and Texas each had one response from a non-accredited facility.

#### Hearing Dogs, Diabetic Alert Dogs, Seizure Alert/Response Dogs, and “Other”

The survey also sought information about these other roles of service dogs, which accounted for the fifth (hearing, *n* = 109 dogs), sixth (diabetic, *n* = 69 dogs), and seventh (seizure, *n* = 11 dogs) most numerous placements in North America for accredited organizations, respectively. For reporting non-accredited facilities, 17 hearing dogs, 30 diabetic alert dogs, and 18 seizure alert/response dogs were placed. The “Other” category was used by only one responding organization that had placed one alert dog for a mast cell disease.

### Non-responding Accredited Facilities

Despite considerable effort to solicit responses from all ADI and/or IGDF accredited facilities, 44 accredited or candidate facilities located in 23 states and 2 provinces did not respond. The numbers of responding and non-responding accredited facilities located in each state or province are listed in Table 1. The responding and non-responding ADI and/or IGDF accredited facilities also are indicated in Figure 5. Additionally, the non-responding facilities are shown in Figure 6, as well as the specific roles of dogs placed by these facilities, as currently indicated in their 2018 websites. These are shown by letter abbreviations for each role, listed in the order used in the original web survey that was provided to facilities.

**Table 1:** Numbers of responding and non-responding ADI/IGDF facilities for the 33 states of the US and the four provinces of Canada with accredited facilities.

**State**	**Responding**	**Non-responding**	**Total**
Alberta	1	0	1
British Columbia	2	0	2
Ontario	2	3	5
Quebec	0	1	1
Alaska	0	1	1
Arizona	0	2	2
California	9	7	16
Colorado	1	1	2
Connecticut	0	1	1
Florida	4	2	6
Hawaii	2	2	4
Illinois	1	0	1
Indiana	0	1	1
Kansas	0	1	1
Kentucky	0	1	1
Maryland	2	0	2
Massachusetts	2	0	2
Michigan	2	0	2
Minnesota	3	1	4
Mississippi	1	0	1
Missouri	1	1	2
New Hampshire	0	1	1
New Jersey	0	1	1
New Mexico	0	2	2
New York	3	2	5
North Carolina	2	2	4
North Oakota	0	2	2
Ohio	3	0	3
Oregon	0	4	4
Pennsylvania	3	2	5
South Carolina	1	0	1
Tennessee	1	0	1
Texas	3	1	4
Virginia	3	0	3
Washington	2	1	3
Wisconsin	1	1	2
Wyoming	1	0	1
Totals	56	44	100

Considering all ADI and/or IGDF facilities, both responding and non-responding, 17 states (34%), primarily in the Southeast, Midwest, and Northeast—Alabama, Arkansas, Delaware, Georgia, Idaho, Iowa, Louisiana, Maine, Montana, Nebraska, Nevada, Oklahoma, Rhode Island, South Dakota, Utah, Vermont, West Virginia (and District of Columbia)—had no facilities that were accredited or candidates of ADI and/or IGDF in 2015. However, among these states, Idaho and Georgia each had a responding non-accredited facility.

In Canada, the only provinces with facilities were British Columbia, Alberta, Ontario, and Quebec.

### Roles of Dogs Related to Accreditation Status of Facilities

The accreditation status of facilities was significantly associated with the roles of dogs they placed (Chi-square: *p* < 0.0001). Hearing, mobility service, and guide dogs, and also autism service dogs, more often were associated with accredited facilities. Diabetes dogs more often were associated with candidate facilities. Non-accredited facilities were associated with psychiatric service and seizure alert dogs.

### Facilities' Accreditation Status and Sources of Dogs Related to Roles of Dogs

The roles of dogs placed by facilities were significantly associated with the facilities' accreditation status and the sources of the dogs placed (Chi-square: *p* < 0.0001). Accredited facilities more often bred their own dogs and used outside breeders, but not clients' pets, shelters or other sources: each source was significantly associated with the facilities' status (Chi-square: *p* < 0.0001). These accredited facilities placed guide, mobility service, autism service and hearing dogs. Seizure alert and diabetes detection dogs somewhat more often were placed by facilities that were candidates for accreditation. Non-accredited facilities often used clients' own pets or dogs from shelters but did not breed their own dogs, and often placed psychiatric service dogs.

### Diversified Roles of Dogs Currently Placed by Facilities

The responding 11 accredited guide dog facilities that were established in the 1930s through 1940s are continuing to place primarily guide dogs, except for one that also placed some dogs trained for other roles. The 23 responding, accredited facilities established 1975–1999 were training dogs for various single roles. For 19 of these facilities, most dogs were placed for the role of mobility service. One facility placed most dogs for autism service, 2 facilities most dogs for guiding, and 1 most dogs for assisting with seizures. Of these, only 6 facilities placed only dogs of one type for one specific disability. This pattern of diversifying to train dogs of a few different role types has continued for the 24 facilities established from 2000 on, with most facilities training dogs of several types to fill various single roles; only 8 facilities placed only one type of dog to address one specific disability.

As mentioned, responding facilities trained dogs with each dog filling only a specific single role for a specified disability. However, most facilities focused on more than one role, e.g., placing various types of dogs to accommodate the needs of persons with visual, hearing, mobility, psychiatric, autistic, seizure, or diabetes disabilities. Among accredited and non-accredited facilities that responded, a majority trained dogs of one or two types, focusing on either one or two specific disabilities. Nonetheless, among responding accredited facilities, 8 trained dogs for 3 single roles, 5 for 4 roles, and 2 for 5 roles. Non-responding ADI facilities similarly listed a median and mode of 2 roles for which they placed dogs; 2 trained dogs for 3 single roles, 4 for 4 roles, 2 for 5 roles, 2 for 6 roles, and 1 for 7 roles. Among non-accredited facilities, 3 trained dogs for 3 single roles, 1 for 4 roles, 3 for 6 roles, and 1 for 7 roles. Both accredited and non-accredited providers are diversifying and placing dogs that accommodate varied specific disabilities. For persons with multiple disabilities, the dog would be trained first to assist with the primary disability, and further personalized training could be developed later.

### Limitations

These data reveal the availability in states and provinces of dogs trained by responding ADI or IGDF facilities and suggest that this may pose an inconvenience for some people seeking an assistance dog. For example, one-third of states lacked an accredited facility. However, almost half of accredited facilities failed to respond and their data are not included in the data presented here. The non-responding could particularly have affected data for facilities placing guide dogs; while relatively few in number, the guide dog facilities often place numerous dogs.

The inconvenience of not having facilities in some states is mitigated by that fact that some facilities provide travel funds that assist prospective handlers of assistance dogs. The study did not explore the extent to which the geographic constraints are inconvenient for people acquiring assistance dogs. Nor was any information collected from dog handlers concerning other possible factors making it inconvenient to acquire a dog.

Surveying non-accredited facilities posed particular challenges. While mentions were found on-line for over one hundred non-accredited facilities, only a small minority of these facilities responded. A high number of kicked-back messages indicated some turnover in these facilities. Some of the responding non-accredited facilities were placing large numbers of dogs. The data on non-accredited facilities obviously are incomplete and not representing all non-accredited facilities, yet the data reveal statistically significant patterns in the roles of dogs placed and the sources of the dogs when the accredited facilities are compared with the non-accredited facilities.

## Discussion

When facilities initially were established and began training and placing guide, hearing, and service dogs, training at each facility was somewhat standardized with a goal of each dog filling certain tasks for its role. Over one hundred tasks are delineated for guide, hearing, and (mobility) service dogs in a document posted by International Association of Assistance Dog Partners [IAADP: (24)]. For example, mobility service dogs are taught many tasks that are: retrieving, carrying (non-retrieval), deposit-based, tug-based, nose nudge-based, pawing-based, bracing-based, and harness-based. While much of the training for guide or mobility service dogs, in terms of tasks, understandably remains consistent, the broadening types of dogs are leading to new lists of required tasks that increasingly become tailored to the particular needs of the person becoming the dog's partner. Facilities in the U.S. and Canada have responded to the personalized needs of their clients by adding new roles for the dogs they place. A strong majority of facilities responding from the U.S. and Canada train and prepare dogs addressing the needs of clients with varied disabilities; for example, they do not only train mobility dogs, but also may train some other dogs for assistance with autism. A similar pattern was reported in Europe, but not internationally in other countries, where facilities more typically still place dogs of only one type (5).

The U.S. has led the way in developing many of the new uses for assistance dogs. With its relaxed laws and enforcement regarding assistance dogs, the U.S. can be the most innovative country in terms of uses and tasks of dogs. Although many states have facilities placing dogs to fill various roles, 11 states with accredited facilities failed to respond and 15 states lacked either an accredited facility or a non-accredited responding facility. In addition to dealing with disabilities that make traveling difficult and inconvenient, some potential partners of assistance dogs face economic challenges due to low incomes (25). Responses in this study reveal that availability for obtaining a well-trained assistance dog is less accessible in some states and provinces than others. Despite the U.S. having numerous facilities that place a large number of assistance dogs, many people in the U.S. have inconvenient access to providers of these dogs. These data expose the geographic hurdles that people with disabilities can face when they consider applying for an assistance dog. The facilities are not evenly distributed throughout the U.S. and Canada, and access to ADI-accredited facilities that train and place assistance dogs can be extremely inconvenient. Needing to negotiate with a distant facility and then travel there for a team training of a few weeks may pose an insurmountable burden for someone seeking an assistance dog. Such persons may become vulnerable targets to corrupt claims by people selling dogs that are sold as well-trained assistance dogs, but the dogs sometimes do not perform as promised.

Guide dogs assure physical safety for partners, as well as assisting with various tasks. This poses special difficulties and hard choices when an assistance dog needs to be retired. Very often the partner needs to quickly begin working with a new dog while deciding at the same time how to retire the older dog, so as to maintain function and travel in the world (26, 27). With only few widely dispersed facilities placing guide dogs, these partners face particular hurdles when retiring a dog is necessary. The waitlist for a dog may be long and the geographic distance may be a further consideration.

Numerous facilities, both accredited and non-accredited, train and place mobility service dogs; this means obtaining one of these dogs may be less challenging than for some other roles. Nonetheless, some outstanding facilities have long wait lists, which can lead people to approach facilities that are not non-profit or that may place less well-trained dogs, or that may offer less follow-up support. Potential handlers face difficult choices when deciding on which facility to focus their efforts.

Dogs that assist with autism and psychiatric disabilities are two newer types of dogs where placements, while still fewer in number than guide and mobility service dogs, are rapidly expanding. Autism dogs are commonly accepted, particularly because they assist with children who have autism. These dogs increasingly are placed by both the accredited and the non-accredited facilities. Acceptance of and demand for psychiatric dogs have increased due to the frequency of post-traumatic stress disorder in veterans. Non-veterans also are seeking psychiatric service dogs to assist with multiple psychiatric disabilities. While relatively few accredited organizations have filled this need for psychiatric service dogs, non-accredited facilities in the U.S. have increased and produce dogs to meet this need. A challenge for these veterans is that the Veterans Administration (VA) only supports people acquiring psychiatric service dogs trained by ADI accredited organizations (20). People needing VA support need to be on a long waiting list for an ADI-trained dog, even though there are many non-accredited facilities providing psychiatric service dogs. Many of these non-accredited facilities are non-profits that are preparing to apply for accreditation. They often assign the handler a dog selected from a shelter and then work with the new handler and dog over a period of about a year [e.g., Animal Rescue Foundation, Pets and Vets (28); Operation Freedom Paws (29)], or place the dog after considerable preparatory training for the new handler [e.g., Starfleet Service Dogs (30)]; some may even assist people in training their own dogs. Thus, all breeds and body sizes of dogs are being used to some extent in assisting roles. A study of dogs registered in California as assistance dogs included equal numbers of large and small dogs, and a lesser number of medium sized dogs (6).

Our results show that accredited facilities continue to place primarily guide, hearing, and mobility dogs, plus the newer autism dogs. Candidate facilities were placing diabetes detection dogs, and non-accredited facilities were likely to place seizure detection and psychiatric service dogs. The accredited facilities placed facility-bred or specially bred dogs, not those from shelters or the handlers' own pet dogs, and the converse was the case for the non-accredited facilities.

Assistance dogs provide life-changing benefits for their handlers. This is widely understood with regards to guide dogs (31), and perhaps also mobility dogs (32). In addition, the full-time assistance dogs of other types also provide essential support of great value to their handlers (33), for example, including dogs for autism (34) or diabetes detection (35). Veterans living with their assistance dogs gain physiological and behavioral benefits (36). Even dogs with no special assistance training can alleviate mental illness symptoms (37). As most readers will recognize, these assistance dogs may facilitate the social interactions their handlers have with members of the public (38).

A problem sometimes experienced by assistance dog handlers is mistreatment of their dogs by the public, such as aggression from other dogs; this even can require early retirement of the dog (26). It undoubtedly impacts handlers that states greatly vary in the legal protection they provide to assistance dogs, ranging from no laws, to civil violations, misdemeanors, or felonies, as maximal penalties. A few of the states with no accredited facilities, Iowa, Montana, West Virginia, as well as District of Columbia, also have no laws protecting service dogs; Connecticut, Maine, and Vermont have only passed civil violations (39). A prospective handler living in a large state like Montana is highly disadvantaged when seeking an assistance dog: no accredited facilities and no legal protection for the dog.

Assistance dog facilities can play a major role in placing assistance dogs from reputable sources and that are adequately trained. ADI accredited facilities are required to be non-profit, and they can be one source of information, as can many of the non-accredited facilities that have a strong track record. The data clarify that non-profit accredited facilities typically follow a more conventional pattern of selecting dogs of known breed history and having an extended training, especially for roles in assisting with guiding, mobility, hearing, and autism. The less formalized non-accredited facilities often use dogs from shelters or the persons' own dogs for training, especially for roles as psychiatric service dogs.

Despite the rapid expansion of assistance dog facilities in the U.S. and Canada, there are significant gaps in the geographic distribution of these providers. This likely creates considerable hardships for many prospective assistance dog partners. Their disabilities and reduced economic status can combine with geographic hurdles as barriers to acquiring an assistance dog—one that could ameliorate some of their challenges with disabilities. Nonetheless, a majority of states had responses from either a mobility or guide dog facility, and many facilities addressed a variety of disabilities with the dogs they placed.

## Ethics Statement

The study included only census information from assistance dog facilities on the numbers of dogs they placed. The study involved no direct involvement with the dogs or handlers, and thus no ethical review was required. We contacted the assistance dog facilities to acquire information on the dogs they had recently placed.

## Author Contributions

SW conducted all electronic communications with survey participants, managed the data for analyses, and prepared the figures. AT assisted in planning the study. NW provided statistical guidance. MY participated in the initial concept and survey design, and assisted in the initial manuscript draft. LH conceived the idea, oversaw data collection, and drafted the manuscript. All authors reviewed all drafts.

### Conflict of Interest

The authors declare that the research was conducted in the absence of any commercial or financial relationships that could be construed as a potential conflict of interest.

## Supplementary Material

The complete dataset is available on Figshare: doi: 10.6084/m9.figshare.7560380
