# Supplementary material for: Geographic Availability of Assistance Dogs: Dogs Placed in 2013–2014 by ADI- or IGDF-Accredited or Candidate Facilities in the United States and Canada, and Non-accredited U.S. Facilities
Source: Front Vet Sci. 2019 Oct 9;6:349. doi: 10.3389/fvets.2019.00349 (PMC6794437; doi:10.3389/fvets.2019.00349)
Supplement: Supplementary file 1 [file Table_1.docx]

Supplementary Table for Figure 1. Placements of Guide Dogs by Responding Facilities in 2013-2014

| **Country** | **State/**  **Province** | **No. of Accredited Facilities** | **No. of Dogs Placed at Accredited Facilities** | **No. of Unaccredited Facilities** | **No. of Dogs Placed at Unaccredited Facilities** |
| --- | --- | --- | --- | --- | --- |
| Canada | Alberta | 1 | 1-5 | 0 | -- |
|  | British Columbia | 1 | 6-15 | 0 | -- |
| United States | California | 1 | 91-200 | 1 | 6-15 |
|  | Florida | 1 | 91-200 | 0 | -- |
|  | Illinois | 1 | 1-5 | 0 | -- |
|  | Michigan | 1 | 201+ | 0 | -- |
|  | New York | 2 | 201+ | 0 | -- |
|  | Texas | 1 | 6-15 | 0 | -- |
|  | Wisconsin | 1 | 6-15 | 0 | -- |
|  | Wyoming | 1 | 1-5 | 0 | -- |

Supplementary Table for Figure 2. Placements of Mobility Dogs by Responding Facilities in 2013-2014

| **Country** | **State/**  **Province** | **No. of Accredited Facilities** | **No. of Dogs Placed at Accredited Facilities** | **No. of Unaccredited Facilities** | **No. of Dogs Placed at Unaccredited Facilities** |
| --- | --- | --- | --- | --- | --- |
| Canada | Alberta | 1 | 6-15 | 0 | -- |
|  | British Columbia | 1 | 1-5 | 0 | -- |
|  | Ontario | 1 | 1-5 | 0 | -- |
| United States | California | 6 | 100+ | 3 | 16-50 |
|  | Colorado | 1 | 16-50 | 0 | -- |
|  | Florida | 3 | 51-100 | 3 | 16-50 |
|  | Georgia | 0 | -- | 1 | 1-5 |
|  | Hawaii | 2 | 1-5 | 0 | -- |
|  | Idaho | 0 | -- | 1 | 16-50 |
|  | Illinois | 1 | 6-15 | 1 | 1-5 |
|  | Indiana | 0 | -- | 1 | 1-5 |
|  | Maryland | 2 | 16-50 | 0 | -- |
|  | Massachusetts | 2 | 100+ | 0 | -- |
|  | Michigan | 1 | 51-100 | 0 | -- |
|  | Minnesota | 3 | 16-50 | 0 | -- |
|  | Mississippi | 1 | 1-5 | 0 | -- |
|  | Missouri | 1 | 1-5 | 1 | 6-15 |
|  | New York | 2 | 51-100 | 0 | -- |
|  | North Carolina | 2 | 6-15 | 0 | -- |
|  | Ohio | 3 | 16-50 | 1 | 1-5 |
|  | Oregon | 0 | -- | 1 | 1-5 |
|  | Pennsylvania | 3 | 6-15 | 1 | 6-15 |
|  | South Carolina | 1 | 1-5 | 0 | -- |
|  | Tennessee | 1 | 6-15 | 0 | -- |
|  | Texas | 1 | 6-15 | 0 | -- |
|  | Virginia | 3 | 16-50 | 1 | 1-5 |
|  | Washington | 2 | 6-15 | 0 | -- |
|  | Wyoming | 1 | 6-15 | 0 | -- |

Supplementary Table for Figure 3. Placements of Autism Service Dogs by Responding Facilities in 2013-2014

| **Country** | **State/**  **Province** | **No. of Accredited Facilities** | **No. of Dogs Placed at Accredited Facilities** | **No. of Unaccredited Facilities** | **No. of Dogs Placed at Unaccredited Facilities** |
| --- | --- | --- | --- | --- | --- |
| Canada | Alberta | 1 | 16-30 | 0 | -- |
|  | British Columbia | 1 | 6-15 | 0 | -- |
|  | Ontario | 1 | 31+ | 0 | -- |
| United States | California | 3 | 31+ | 2 | 6-15 |
|  | Colorado | 1 | 1-5 | 0 | -- |
|  | Connecticut | 0 | -- | 1 | 16-30 |
|  | Florida | 2 | 16-30 | 2 | 6-15 |
|  | Illinois | 1 | 1-5 | 0 | -- |
|  | Indiana | 0 | -- | 1 | 1-5 |
|  | Massachusetts | 1 | 16-30 | 0 | -- |
|  | Minnesota | 2 | 6-15 | 0 | -- |
|  | Mississippi | 1 | 6-15 | 0 | -- |
|  | Ohio | 1 | 1-5 | 1 | 1-5 |
|  | Oregon | 0 | -- | 1 | 16-30 |
|  | Pennsylvania | 3 | 6-15 | 1 | 1-5 |
|  | South Carolina | 1 | 1-5 | 0 | -- |
|  | Virginia | 2 | 6-15 | 0 | -- |

Supplementary Table for Figure 4. Placements of Psychiatric Service Dogs by Responding Facilities in 2013-2014

| **Country** | **State/**  **Province** | **No. of Accredited Facilities** | **No. of Dogs Placed at Accredited Facilities** | **No. of Unaccredited Facilities** | **No. of Dogs Placed at Unaccredited Facilities** |
| --- | --- | --- | --- | --- | --- |
| United States | Arizona | 0 | -- | 1 | 16+ |
|  | California | 2 | 16+ | 2 | 6-15 |
|  | Colorado | 1 | 6-15 | 0 | -- |
|  | Florida | 4 | 16+ | 2 | 16+ |
|  | Georgia | 0 | -- | 1 | 1-5 |
|  | Idaho | 0 | -- | 1 | 6-15 |
|  | Massachusetts | 1 | 6-15 | 0 | -- |
|  | Minnesota | 1 | 1-5 | 0 | -- |
|  | Mississippi | 1 | 1-5 | 0 | -- |
|  | Missouri | 0 | -- | 1 | 6-15 |
|  | New Mexico | 0 | -- | 1 | 16+ |
|  | New York | 1 | 6-15 | 0 | -- |
|  | North Carolina | 1 | 1-5 | 0 | -- |
|  | Ohio | 0 | -- | 1 | 6-15 |
|  | Pennsylvania | 3 | 6-15 | 1 | 1-5 |
|  | South Carolina | 1 | 1-5 | 0 | -- |
|  | Texas | 0 | -- | 1 | 16+ |
|  | Virginia | 1 | 1-5 | 0 | -- |

Supplementary Table for Figure 5. Numbers of Responding Accredited Facilities as Compared with the Total Numbers of Accredited Facilities

| **Country** | **State/**  **Province** | **No. of Responding Facilities** | **Total Facilities** |
| --- | --- | --- | --- |
| Canada | Alberta | 1 | 1 |
|  | British Columbia | 2 | 2 |
|  | Ontario | 2 | 3 |
|  | Quebec | 0 | 1 |
| United States | Alaska | 0 | 1 |
|  | Arizona | 0 | 2 |
|  | California | 9 | 16 |
|  | Colorado | 1 | 2 |
|  | Connecticut | 0 | 1 |
|  | Florida | 4 | 6 |
|  | Hawaii | 2 | 4 |
|  | Illinois | 1 | 1 |
|  | Indiana | 0 | 1 |
|  | Kansas | 0 | 1 |
|  | Kentucky | 0 | 1 |
|  | Maryland | 2 | 2 |
|  | Massachusetts | 2 | 2 |
|  | Michigan | 2 | 2 |
|  | Minnesota | 3 | 4 |
|  | Mississippi | 1 | 1 |
|  | Missouri | 1 | 2 |
|  | New Hampshire | 0 | 1 |
|  | New Jersey | 0 | 1 |
|  | New Mexico | 0 | 2 |
|  | New York | 3 | 5 |
|  | North Carolina | 2 | 4 |
|  | North Dakota | 0 | 2 |
|  | Ohio | 3 | 3 |
|  | Oregon | 0 | 4 |
|  | Pennsylvania | 3 | 5 |
|  | South Carolina | 1 | 1 |
|  | Tennessee | 1 | 1 |
|  | Texas | 3 | 4 |
|  | Virginia | 3 | 3 |
|  | Washington | 2 | 3 |
|  | Wisconsin | 1 | 2 |
|  | Wyoming | 1 | 1 |

Supplementary Table for Figure 6. Non-Responding Accredited Facilities and Roles of Dogs They Place

| **Country** | **State/**  **Province** | **No. of Non-Responding Facilities** | **Roles of Dogs** |
| --- | --- | --- | --- |
|  | Ontario | 3 | GHMSAPD |
|  | Quebec | 1 | GMA |
| United States | Alaska | 1 | M |
|  | Arizona | 2 | HMPD |
|  | California | 7 | GHMSAPD |
|  | Colorado | 1 | M |
|  | Connecticut | 1 | G |
|  | Florida | 2 | AP |
|  | Hawaii | 2 | HMS |
|  | Indiana | 1 | MAPD |
|  | Kansas | 1 | GM |
|  | Kentucky | 1 | M |
|  | Minnesota | 1 | MP |
|  | Missouri | 1 | HM |
|  | New Hampshire | 1 | M |
|  | New Jersey | 1 | G |
|  | New Mexico | 2 | MSAPD |
|  | New York | 2 | GP |
|  | North Carolina | 2 | HMSAPDO |
|  | North Dakota | 2 | MSPD |
|  | Oregon | 4 | HMAP |
|  | Pennsylvania | 2 | MPDO |
|  | Texas | 1 | MP |
|  | Washington | 1 | GHMSAPD |
|  | Wisconsin | 1 | GMAP |
